# Supplementary material for: Instabilities of heavy magnons in an anisotropic magnet
Source: Nat Commun. 2023 Jul 14;14:4199. doi: 10.1038/s41467-023-39940-1 (PMC10349074; doi:10.1038/s41467-023-39940-1)
Supplement: Supplementary file 1 — Supplementary Information [file 41467_2023_39940_MOESM1_ESM.pdf]

Supplementary Information

Supplementary Figure 1: Exchange Interactions

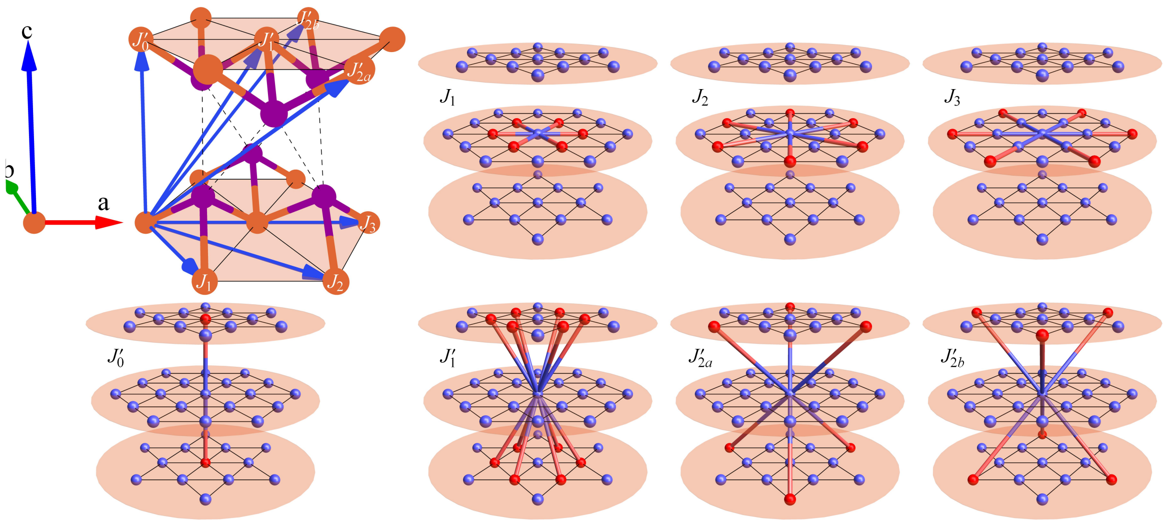

Supplementary Figure 1. Magnetic exchange interactions in FeI_2.

Supplementary Figure 2: Magnetic Structure

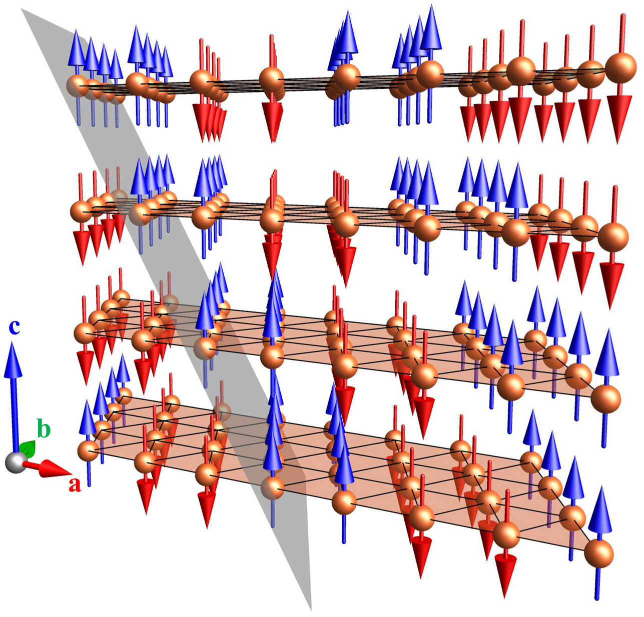

Supplementary Figure 2. Magnetic structure of FeI_2.

Supplementary Figure 3: Bragg Diffraction in Field

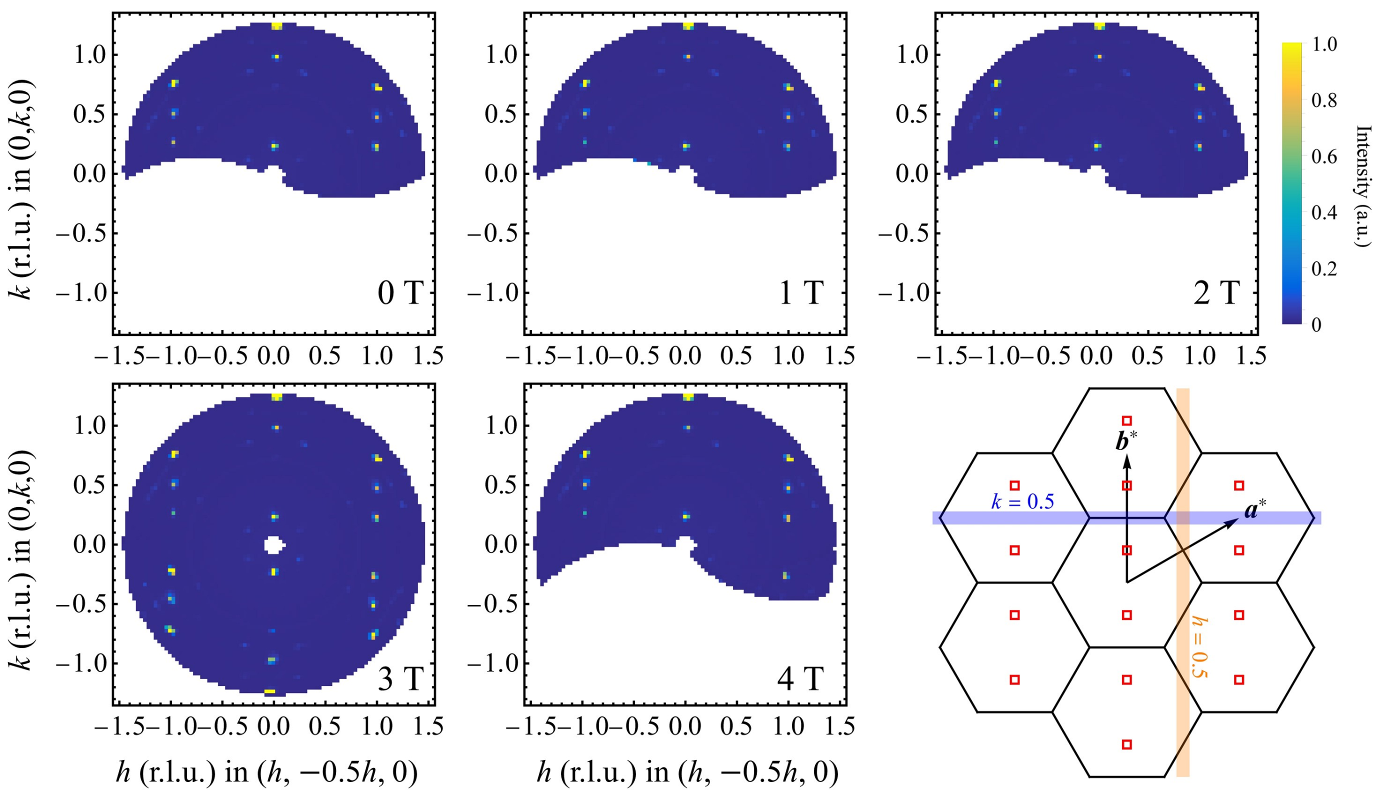

Supplementary Figure 3. Structural and magnetic Bragg reflections of FeI_2 in applied magnetic fields within the AF phase. Elastic cuts $(-0.2 \leq E \leq 0.2$ meV) through neutron scattering data with integrated out-of-plane momenta $(-0.3 \leq l \leq 0.3$ r.l.u.). The sample was first cooled down through T_{N} to $T = 1.8$ K in zero field where preliminary data was collected, showing that magnetic Bragg peaks associated with all three magnetic domains of $\boldsymbol{k}_{\mathrm{AF}}$ co-existed in the sample. The magnetic field was then increased to $\mu_0 H = 3$ T where reflections corresponding to two out of the three domains are strongly suppressed. Magnetic Bragg peaks associated with the propagation vector $\boldsymbol{k}_{\mathrm{AF}}^{(1)} = (0, 1/4, 1/4)$ are dominant, indicating that a predominantly single-domain magnetic structure is stabilized in the sample. The selection of a single domain by magnetic fields results from two fortuitous factors – the tilting of magnetic moments with respect to the easy-axis produced by off-diagonal exchange interactions [24] and a small (unintentional) out-of-plane misalignment ($\lesssim 5°$) in the sample mount. The single-domain magnetic state is maintained after lowering the magnetic field to zero while keeping the sample at $T = 1.8$ K. The red squares in the Brillouin Zone drawings indicates positions of magnetic Bragg peaks projected to the $l = 0$ plane.

Supplementary Figure 4: Field-dependent spectra in the $(h, -0.5h, 0)$-direction

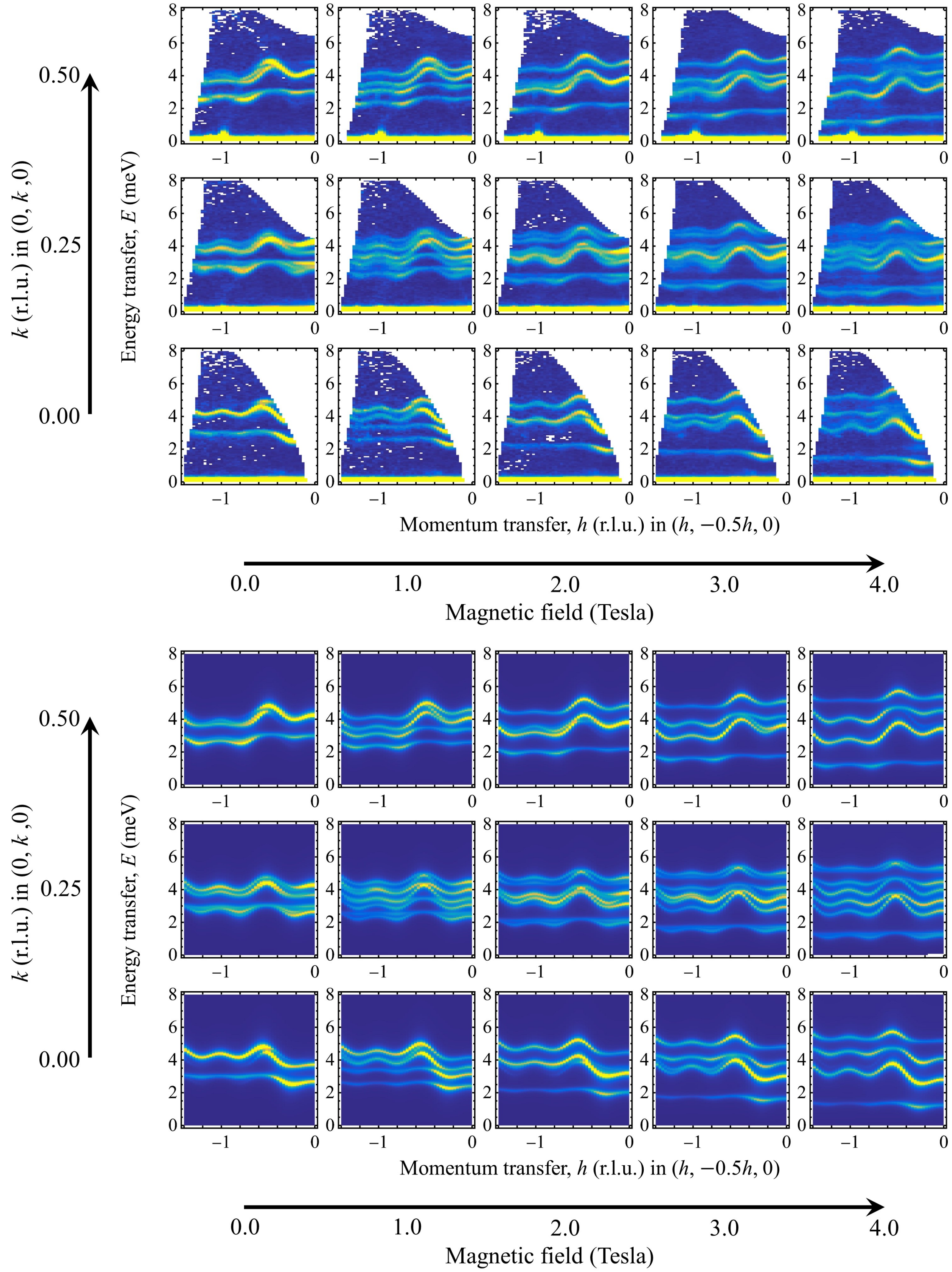

Supplementary Figure 4. Comparison of field-dependent spectra between experiment (top) and GSLW calculations (bottom) for cuts along the $(h, -0.5h, 0)$-direction for $k = 0$, $\frac{1}{4}$ and $\frac{1}{2}$ as indicated and $l = 0$.

Supplementary Figure 5: Field-dependent spectra in the $(0, k, 0)$-direction

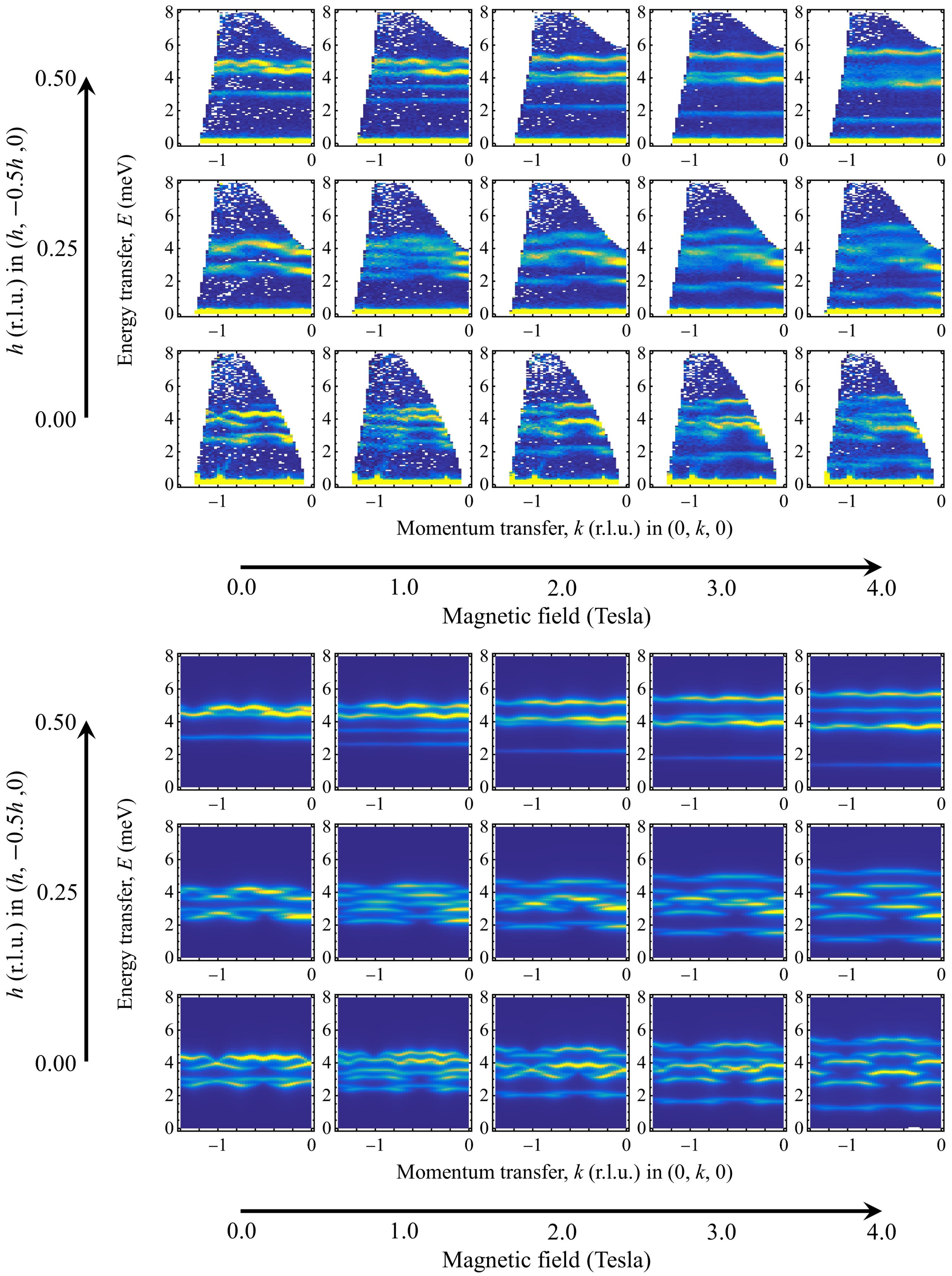

Supplementary Figure 5. Comparison of field-dependent spectra between experiment (top) and GSLW calculations (bottom) for cuts along the $(0, k, 0)$-direction for $h = 0$, $\frac{1}{4}$ and $\frac{1}{2}$ as indicated and $l = 0$.

Supplementary Figure 6: Fitted instrumental lineshape

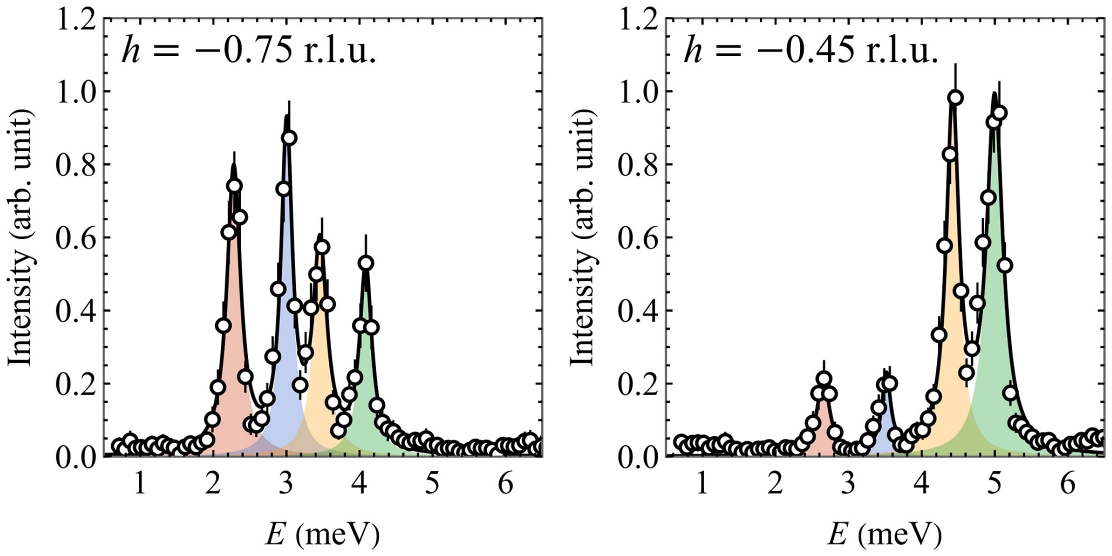

Supplementary Figure 6. Lorentzian peak fitting of spectra at $k = -0.5$ and $\mu_0 H = 1\,\mathbf{T}$, serving as experimental calibration of instrumental resolution for band bottom ($h = -0.75$) and band top ($h = -0.45$). See Tab. S1 for fitted parameters.

Supplementary Table 1: Fitted parameters of inelastic spectra

$\mu_0 H = 1\,\mathrm{T}$, $k = 0.5\,\mathrm{r.l.u.}$, $h = -0.75\,\mathrm{r.l.u.}$

E_0 (meV)	2.28(3)	3.00(1)	3.46(2)	4.09(3)
FWHM (meV)	0.23(3)	0.18(1)	0.23(1)	0.20(3)

$\mu_0 H = 1\,\mathrm{T}$, $k = 0.5\,\mathrm{r.l.u.}$, $h = -0.45\,\mathrm{r.l.u.}$

E_0 (meV)	2.65(3)	3.52(2)	4.42(7)	5.00(2)
FWHM (meV)	0.20(8)	0.14(3)	0.21(1)	0.27(2)

$\mu_0 H = 3\,\mathrm{T}$, $k = 0.5\,\mathrm{r.l.u.}$, $h = -0.75\,\mathrm{r.l.u.}$

E_0 (meV)	1.55(5)	2.51(9)	2.77(5)	2.98(7)	3.50(2)	3.68(4)	4.67(3)
FWHM (meV)	0.23(4)	0.15(8)	0.24(1)	0.22(2)	0.42(1)	0.17(8)	0.25(5)

$\mu_0 H = 4\,\mathrm{T}$, $k = 0.5\,\mathrm{r.l.u.}$, $h = -0.45\,\mathrm{r.l.u.}$

E_0 (meV)	1.45(2)	3.76(1)	4.41(3)	5.62(1)
FWHM (meV)	0.25(2)	0.38(1)	0.57(1)	0.25(9)

Table S1. Peak center (E_0) and full width at half maximum (FWHM) of Lorentzian fitting of inelastic spectra. The fitted FWHM for spectra at $\mu_0 H = 1\,\mathrm{T}$ are consistent with the nominal instrumental resolution of $\sim 0.2\,\mathrm{meV}$. Peaks with significantly broader widths due to magnon decay are marked red.
